# Trends in intravenous antimicrobial start rates in outpatient hemodialysis centers, United States, 2012–2021

**DOI:** 10.1017/ash.2025.37

**Published:** 2025-03-26

**Authors:** W. Wyatt Wilson, Hannah Hua, Qunna Li, Minn M. Soe, Ibironke W. Apata, Lu Meng, Jeneita M. Bell, Emily McDonald, Jonathan R. Edwards, Sarah Kabbani, Shannon Novosad

**Affiliations:** 1 Division of Healthcare Quality Promotion, Centers for Disease Control and Prevention, Atlanta, GA, USA; 2 Division of Hospital Medicine, Emory School of Medicine, Atlanta, GA, USA; 3 Division of Renal Medicine, Emory School of Medicine, Atlanta, GA, USA

## Abstract

Using National Healthcare Safety Network data, an interrupted time series of intravenous antimicrobial starts (IVAS) among hemodialysis patients was performed. Annual adjusted rates decreased by 6.64% (January 2012–March 2020) and then further decreased by 8.91% until December 2021. IVAS incidence trends have decreased since 2012, including during the early COVID-19 pandemic.

## Introduction

Nearly 1 in 3 persons on maintenance hemodialysis receives intravenous antimicrobials annually, and among those administered, up to 50% may not be clinically indicated.^1^ During the early COVID-19 pandemic, antibiotic use rates increased in US hospitals,^2^ raising concern that similar effects would occur in other healthcare settings like dialysis clinics. A previous analysis of National Healthcare Safety Network (NHSN) data found that pooled annual rates of intravenous antimicrobial starts (IVAS) reported from 2016 to 2020 in outpatient hemodialysis clinics decreased.^3^ However, this analysis did not account for granular trends in antibiotic prescribing or effects from the early COVID-19 pandemic.

To further characterize outpatient IVAS among persons receiving outpatient hemodialysis, we analyzed trends of IVAS rates reported to NHSN from 2012 to 2021, controlling for seasonal patterns, facility characteristics, and the COVID-19 pandemic.

## Methods

NHSN is a US surveillance system that tracks healthcare-associated infections across ≥38,000 facilities. Since January 2012, outpatient hemodialysis facilities follow a protocol and report monthly data to participate in the End Stage Renal Disease Quality Incentive Program, a value-based purchasing program administered by the Centers for Medicare and Medicaid Services.^4^ Facilities submit an annual Facility Practices Survey, a monthly Facility Census Form, which records the number of patients on the first 2 workdays of the month and provides patient-months for point incidence rate calculations, and a monthly Dialysis Event Form. There are 3 reportable dialysis events: an IVAS, a positive blood culture bloodstream infection (BSI), and a pus, redness, or swelling event at the vascular access site.

IVAS data reported to NHSN during 2012–2021 were analyzed. To be considered a new IVAS event, at least 21 days had to elapse following another IVAS event. Events were excluded if they were duplicative, violated the 21-day rule, did not have a vascular access type specified, or were from a facility reporting missing or zero patient-months for that month. IVAS events and the monthly number of dialysis patients for each facility were summarized by vascular access type according to infection risk (central venous catheters (CVC), other (eg, catheter-graft hybrids), arteriovenous grafts (AVG), and arteriovenous fistula (AVF) access) to calculate crude incidence rates.

An interrupted time series with mixed effects negative binomial regression modeled the trend of IVAS rates associated with the early COVID-19 pandemic, March 2020–December 2021. The dependent variable was monthly IVAS events at each facility, offset by the natural logarithm of the facility patient-months. The independent variables were a linear term for months, a binary indicator variable for before and after March 2020, and an interaction term between these 2 variables, which allowed an estimate of any level change of IVAS rates in March 2020. Other covariates included were vascular access type, seasonality (expressed as sine (sin(2*π*month/12)) and cosine (cos(2*π*month/12)) to predict yearly incidence peak and nadir^5^) and facility characteristics, including station number in quartiles, belonging to a dialysis organization, and hospital affiliation. A sensitivity analysis was conducted for facilities reporting to NHSN for at least 6 continuous months per year from 2012 to 2021. Analyses were performed using SAS software (version 9.4; SAS Institute).

## Results

The number of outpatient hemodialysis facilities reporting to NHSN increased from 5,581 in 2012 to 7,313 in 2021. Facility characteristics remained constant throughout the study: approximately 87.39% were not associated with a hospital, and 88.70% were part of a dialysis organization. The median number of dialysis stations was 17 (interquartile range: 12, 24).

**Figure 1.**
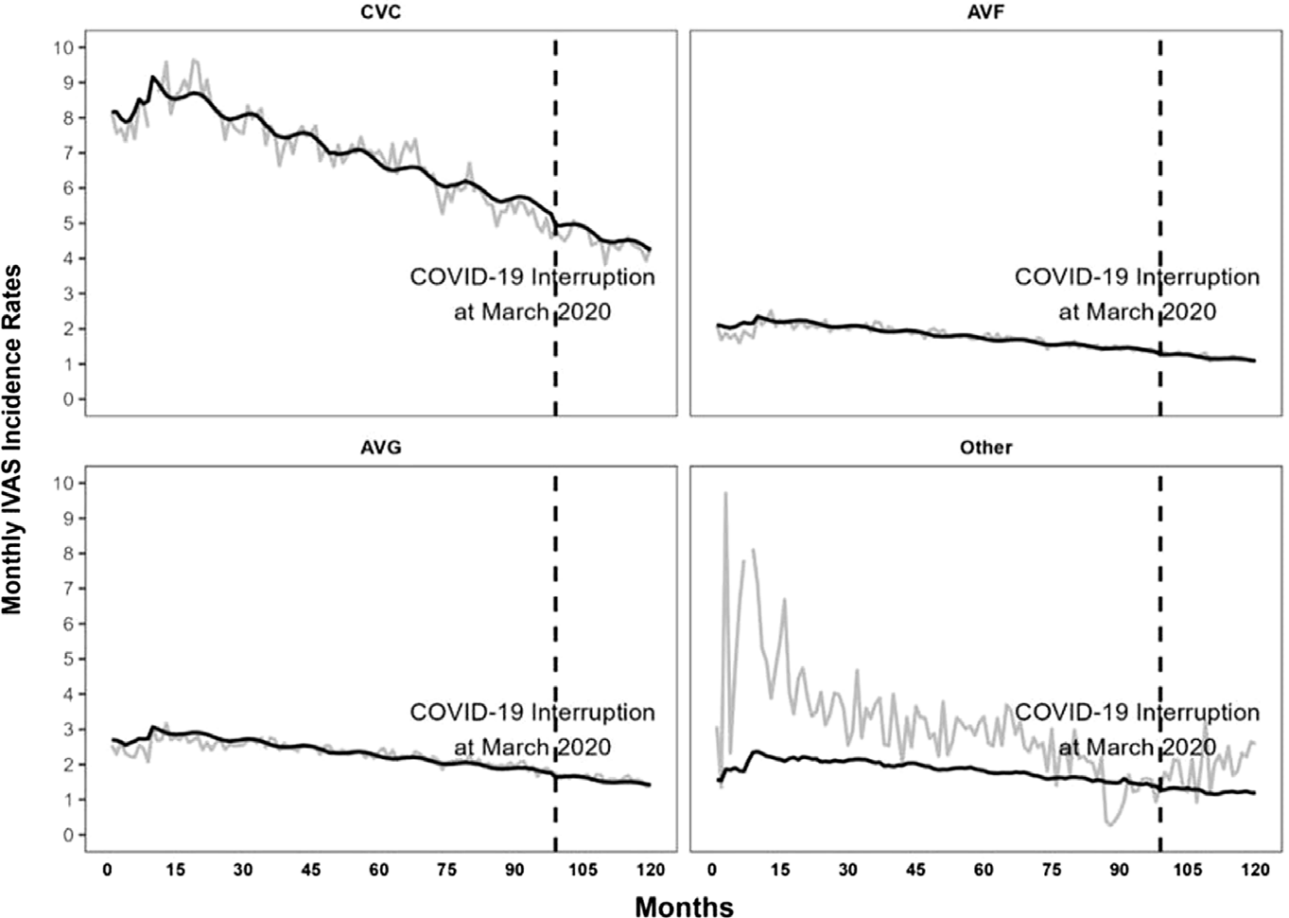
Monthly crude and predicted intravenous antimicrobial starts (IVAS) per 100 patient-months by vascular access type, National Healthcare Safety Network, 2012–2021. CVC, central venous catheter; AVF, arteriovenous fistula; AVG, arteriovenous graft.

Crude IVAS rates decreased annually from 2012 to 2021. Patients with CVC, other, AVG, and AVF access had IVAS rates of 8.86, 6.03, 2.70, and 2.12 events per 100 patient-months in 2012, which decreased to 4.31, 2.19, 1.55, and 1.16 events per 100 patient-months, respectively, in 2021 (Figure 1). Between 2012 and February 2020, the pooled crude IVAS rate was 2.90 events per 100 patient-months (1,060,504/36,613,799), which was 6.92, 2.96, 2.33, and 1.80 events per 100 patient-months, respectively, when stratified by CVC, other, AVG, and AVF access. During the early COVID-19 period, the pooled crude IVAS rate was 2.07 events per 100 patient-months (195,717/9,455,621) and 4.49, 1.96, 1.61, and 1.22 events per 100 patient-months when stratified by vascular access route.

Adjusted IVAS rates decreased by an average of 6.64% each year (95% CI, 6.56%–6.73%) from January 2012 to February 2020 (Table 1). In March 2020, there was a 5.85% drop (95% CI, 4.85%–6.84%) in average annual IVAS rates. During the early COVID-19 period, the trend of decreasing annual IVAS rates was sustained at 8.91% (95% CI, 8.10%–9.74%).

**Table 1.** Intravenous antimicrobial starts interrupted time series model with estimated adjusted incidence rate ratios (aRR) and annual percent change, National Healthcare Safety Network, 2012–2021

**Model parameter** ^a^	**Parameter estimates at monthly level**	**Yearly aRR** **(95% CI)**	**Percent change per year** ^b^ **(95% CI)**	***P*** **-value**
**Time trend before COVID-19, Jan 2012–Feb 2020 (β1)** ^c^	−0.006	0.934 (0.933, 0.934)	−6.645 (−6.735, -6.555)	<0.001
**Change in slope during COVID-19, Mar 2020–Dec 2021 (β2)** ^c^	−0.002	0.976 (0.967, 0.985)	−2.430 (−3.316, −1.548)	<0.001
**Time trend during COVID-19, Mar 2020–Dec 2021 (β1+ β2)** ^c^	−0.008	0.911 (0.903, 0.919)	−8.913 (−9.745, −8.097)	<0.001
	**Parameter estimates**	**aRR (95% CI)**	**Percent change** ^b^	***P*** **-value**
**Level change of COVID-19 interruption in March 2020**	−0.060	0.941 (0.932, 0.951)	−5.854 (−6.843, −4.853)	<0.001
**Vascular access types**				
Central venous catheter (CVC)	1.384	3.991 (3.974, 4.008)	–	<0.001
Arteriovenous graft (AVG)	0.261	1.298 (1.290, 1.305)	–	<0.001
Other dialysis access	0.027	1.027 (0.987, 1.070)	–	0.201
Arteriovenous fistula (AVF)	Ref	Ref	–	–
**Hospital affiliation**				
Free-standing^d^	0.023	1.023 (1.004, 1.042)	–	0.017
Hospital	Ref	Ref	–	–
**Group member**				
Yes	0.098	1.103 (1.085, 1.122)	–	<0.001
No	Ref	Ref	–	–
**Number of dialysis stations**				
18–24	0.023	1.023 (1.011, 1.036)	–	<0.001
25 or more	0.048	1.049 (1.033, 1.065)	–	<0.001
17 or fewer	Ref	Ref	–	–
**Sine function**	−0.023	–	–	<0.001
**Cosine function**	−0.008	–	–	<0.001

Note. CI, confidence interval.

a Negative binomial mixed model adjusts for vascular access type, seasonal factors (sine: sin (2*π*month/12 and cosine: cos(2*π*month/12)) and location/hospital affiliation.

b Percent change equals (aRR-1)×100.

c Data modeled at facility-month level, aRR and percent change is calculated at yearly level.

d Location could be freestanding or a freestanding location owned by a hospital.

Patients with CVC and AVG were 3.99 (95% CI, 3.97%–4.01%) and 1.30 (95% CI, 1.29%–1.31%), respectively, times as likely to have an IVAS compared with patients with AVF access. Facilities with 25 or more stations were 1.05 (95% CI, 1.03%–1.07%) times as likely to have an IVAS compared with facilities with 17 or fewer stations. Annual IVAS rates peaked in August and reached their nadir in February.

Among 5,244 (71.7%) facilities reporting continuously, adjusted IVAS rates decreased by 6.57% (95% CI, 6.47%–6.66%) annually from 2013 to 2021. There was a discrete drop of 6.15% (95% CI, 5.06%–7.23%) in IVAS rates in March 2020. The IVAS rates decreased by 8.72% (95% CI, 7.8%–9.62%) annually during the early COVID-19 period.

## Discussion

This report uses national surveillance data to demonstrate a decreasing trend in annual IVAS incidence from 2012 to 2021. The downward annual trend was sustained after March 2020, when the World Health Organization declared COVID-19 a pandemic and US states began issuing stay-at-home orders.^6,7^ Persons with CVC access had significantly higher IVAS rates than those with other access types. IVAS rates peaked in August each year, which mirrors seasonal variation patterns of hospital-acquired bloodstream infections, particularly central-line associated BSIs.^8^

A heightened commitment to infection prevention and antimicrobial stewardship during the study period, specifically in persons receiving outpatient hemodialysis, may explain decreasing IVAS rates. In 2009, the Centers for Disease Control and Prevention (CDC) developed the Core Interventions for Dialysis Bloodstream Infections^a^ and, in 2016, created a partnership called the Making Dialysis Safer for Patients Coalition^b^ to prevent hemodialysis-related infections. Both initiatives promoted the implementation of infection prevention practices, including BSI surveillance, hand hygiene, and catheter care practices, which may mitigate the downstream need for antimicrobial therapy, particularly in patients with CVC access, when implemented effectively.

Sustained declines in IVAS rates during the early COVID-19 pandemic coincided with an expert consensus publication by the CDC and the American Society of Nephrology in October 2020, which emphasized strategies decreasing unnecessary antimicrobial prescribing in dialysis centers.^9^ Additionally, in March 2021, the United States prioritized COVID-19 vaccination in persons receiving outpatient maintenance dialysis, resulting in 64.5% of 483,602 persons being partially or fully vaccinated by June 2021.^10^ Social distancing measures, adherence to transmission-based precautions, and high vaccination rates among hemodialysis patients may have led to decreased rates of febrile illness that would have otherwise prompted empiric antimicrobial therapy. At the same time, lower IVAS rates could also be explained by patient avoidance of healthcare settings during the early pandemic when sick, which could disproportionally affect the numerator of IVAS rates.

This study has some limitations. Although facilities have incentives to report to NHSN, reporting consistency and quality may depend on resources and protocol adherence. However, NHSN performs quarterly data validations on random Dialysis Safety Module samples. Although the random effects mixed model accounts for facility number and heterogeneity, a sensitivity analysis of continuous reporters did not find a meaningful impact of unbalanced reporting. This analysis cannot explain the appropriateness of the antimicrobial therapy administered. Additional data that might support a clinical indication for treatment, such as symptoms during the event or whether the IVAS was new versus a continuation from another healthcare setting,^3^ were part of protocol updates during different years across the study period and, thus, not individually analyzed. Furthermore, conventional antimicrobial use measures, including duration of therapy, type of antimicrobial, de-escalation protocols, or prescribing for infection prophylaxis, are not part of the DE form. Until antimicrobial use measures are incorporated into the NHSN DE protocol, understanding the clinical impact of IVAS is limited.

Annual IVAS rates decreased in outpatient hemodialysis centers from 2012 to 2021, including a period of sustained decline during the early COVID-19 pandemic. Increased awareness of infection prevention practices, antimicrobial stewardship, and COVID-19 mitigation measures in dialysis clinics, such as vaccination and transmission-based precautions, may explain enduring trends during the pandemic. Additional stewardship measures in the NHSN DE form might clarify how clinical factors contribute to IVAS incidence and better describe the impact of interventions in dialysis facilities.

## Data Availability

Line-level data are not made publicly available in accordance with the NHSN Agreement to Participate and Consent. Publicly available data are prepared in aggregate on the NHSN website to protect against the identification of patients and healthcare facilities.

## Footnotes

a

https://www.cdc.gov/dialysis-safety/hcp/clinical-safety/index.html

b

https://www.cdc.gov/dialysis-safety/making-dialysis-safer-coalition/index.html

## References

[1] Snyder GM , Patel PR , Kallen AJ , Strom JA , Tucker JK , D’Agata EM . Factors associated with the receipt of antimicrobials among chronic hemodialysis patients. Am J Infect Control 2016;44(11):1269–1274. doi: 10.1016/j.ajic.2016.03.034 27184209 PMC5748878

[2] O’Leary EN , Neuhauser MM , Srinivasan A , et al. Impact of the COVID-19 pandemic on inpatient antibiotic use in the United States, January 2019 through July 2022. Clin Infect Dis 2024;78(1):24–26. doi: 10.1093/cid/ciad453 37536269 PMC11629484

[3] Wilson WW , Gouin KA , Fike L , et al. Intravenous antimicrobial starts among hemodialysis patients in the national healthcare safety network dialysis component, 2016-2020. Kidney360 2023;4(7):971–975. doi: 10.34067/KID.0000000000000167 37257087 PMC10371262

[4] CDC. NHSN and CMS End Stage Renal Dialysis Quality Incentive Program (ESRD QIP) Rule. Accessed April 18, 2024. https://www.cdc.gov/nhsn/faqs/dialysis/faq-esrd-qip.html#q1

[5] Stolwijk AM , Straatman H , Zielhuis GA . Studying seasonality by using sine and cosine functions in regression analysis. J Epidemiol Community Health 1999;53(4):235–238. doi: 10.1136/jech.53.4.235 10396550 PMC1756865

[6] WHO Directors-General’s opening remarks at the media briefing on COVID-19-11 March 2020. March 11, 2020, https://www.who.int/director-general/speeches/detail/who-director-general-s-opening-remarks-at-the-media-briefing-on-covid-19---11-march-2020

[7] State, territorial and county COVID-19 orders and proclamations for individuals to stay home. March 15, 2020, https://data.cdc.gov/Policy-Surveillance/U-S-State-and-Territorial-Stay-At-Home-Orders-Marc/y2iy-8irm/about_data

[8] Blot K , Hammami N , Blot S , Vogelaers D , Lambert ML . Seasonal variation of hospital-acquired bloodstream infections: a national cohort study. Infect Control Hosp Epidemiol 2022;43(2):205–211. doi: 10.1017/ice.2021.85 33975668

[9] Apata IW , Kabbani S , Neu AM , et al. Opportunities to improve antibiotic prescribing in outpatient hemodialysis facilities: a report from the American Society of Nephrology and Centers for Disease Control and Prevention antibiotic stewardship white paper writing group. Am J Kidney Dis 2021;77(5):757–768. doi: 10.1053/j.ajkd.2020.08.011 33045256 PMC7546947

[10] Patel PR , Tanz LJ , Hamilton E , et al. Assessment of provision of COVID-19 vaccination in dialysis clinics and patient vaccination coverage. JAMA Intern Med 2022;182(6):676–678. doi: 10.1001/jamainternmed.2022.0627 35377396 PMC8981065

